# Progressivity of out-of-pocket costs for Medicare-subsidised services and medicines in Australia.

**DOI:** 10.23889/ijpds.v7i3.1976

**Published:** 2022-08-25

**Authors:** Hsei Di Law, Dinith Marasinghe, Nicholas Biddle, Danielle Butler, Emily Lancsar, Jane Desborough, Rosemary Korda

**Affiliations:** 1 Australian National University

## Objectives

In line with affordability and equity principles, Medicare—Australia’s universal public health insurance system—has measures to limit out-of-pocket costs (OOPC), especially among lower income households. We examined the distribution of OOPC for Medicare-subsidised out-of-hospital services and prescription medicines, for Census households, according to their ability to pay.

## Approach

We used 2016 Australian Census data linked to Medicare claims to obtain OOPC for out-of-hospital services and medicines in each household in 2017-18. We derived household disposable income by combining income information from the Census linked to income tax and social security data. All data were available from the Multi-Agency Data Integration Project, enabled through a partnership of various government agencies. We quantified OOPC as a proportion of equivalised household disposable income and calculated Kakwani indices (K) to measure progressivity. We also used linked National Health Survey data to analyse costs separately by chronic conditions.

## Results

We analysed 85% (n=6,830,365) of all Census private households. Overall, OOPC as a percentage of equivalised household disposable income decreased from 1.16% (out-of-hospital services) and 1.35% (prescription medicines) in the poorest decile to 0.63% and 0.34% in the richest decile, respectively. The regressive trend was less pronounced for out-of-hospital services (K = -0.06), with percentage OOPC relatively stable between the 2nd and 9th income deciles; while percentage OOPC decreased steeply with increasing income for medicines (K = -0.24). (Chronic conditions results will be presented—embargoed at time of submission).

## Conclusion

OOPC for out-of-hospital Medicare services were mildly regressive while those for prescription medicines were distinctly regressive. Actions to reduce inequity in OOPC for medicines, such as reducing the co-payments for low income households should be considered.

